# Iterative High-Accuracy Parameter Estimation of Uncooperative OFDM-LFM Radar Signals Based on FrFT and Fractional Autocorrelation Interpolation

**DOI:** 10.3390/s18103550

**Published:** 2018-10-19

**Authors:** Yifei Liu, Yuan Zhao, Jun Zhu, Ying Xiong, Bin Tang

**Affiliations:** School of information and Communication Engineering, University of Electronic Science and Technology of China, Chengdu 611731, China; zy_uestc@outlook.com (Y.Z.); uestczhujun@163.com (J.Z.); Xiongy@uestc.edu.cn (Y.X.); BinT@uestc.edu.cn (B.T.)

**Keywords:** uncooperative sensor signal processing, MIMO radar, fractional Fourier transform, fractional autocorrelation interpolation

## Abstract

To improve the parameter estimation performance of uncooperative Orthogonal Frequency Division Multi- (OFDM) Linear Frequency Modulation (LFM) radar signals, this paper proposes an iterative high-accuracy method, which is based on Fractional Fourier Transform (FrFT) and Fractional Autocorrelation (FA) interpolation. Two iterative estimators for rotation angle and center frequencies are derived from the analytical formulations of the OFDM-LFM signal. Both estimators are designed by measuring the residual terms between the quasi peak and the real peak in the fractional spectrum, which were obtained from the finite sampling data. Successful elimination of spectral leakage caused by multiple components of the OFDM-LFM signal is also proposed by a sequential removal of the strong coefficient in the fractional spectrum through an iterative process. The method flow is given and its superior performance is demonstrated by the simulation results.

## 1. Introduction

As a novel radar system, the Multiple-Input Multiple-Output (MIMO) radar employs multiple transmitting antennas to emit mutually orthogonal waveforms and uses multiple receiving antennas to process the echo signals simultaneously [1]. Subject to current technical conditions, the coherent MIMO radar technique is commonly used in modern MIMO radar systems [2].

In this paper, we focus on the high-accuracy parameter estimation of uncooperative Orthogonal Frequency Division Multi- (OFDM) Linear Frequency Modulation (LFM) signals, which have been widely used in coherent MIMO radar systems. In the past decades, much research has been conducted on OFDM-LFM waveform design [1,2,3]. However, only a few studies have discussed parameter estimation for uncooperative OFDM-LFM signals in electronic warfare systems. The signal model of the intercepted MIMO signals based on a single-channel receiver has been analyzed in the literature [4,5]. Moreover, an improved Multiple Wigner–Hough Transform (MWHT) [6] was proposed to enhance the performance of signal detection and parameter estimation for OFDM-LFM signals. Other estimation algorithms based on likelihood estimators or optimization methods for multicomponent LFM signals were applied in [7,8,9]. However, these earlier algorithms have limitations on the estimation accuracy and efficiency due to the cross-term and picket fence effects. Besides, most of these algorithms also lack computational efficiency, making them more difficult and expensive to realize [10].

Based on the fast algorithm of Fractional Fourier Transform (FrFT) that was proposed by Ozaktas [11], related research works [12,13,14,15,16,17] have brought the application of digital FrFT (DFrFT) to maturity. On the one hand, fractional derivatives and calculus in a complex plane were studied in [13,14,15,16], which are beneficial in establishing fractional models in engineering. Furthermore, the contributions in [17] proposed the fractional geometric calculus and extended the fractional calculus to any dimension. On the other hand, the analytical FrFT formulations of multicomponent LFM signals were introduced in [10]. Conventional DFrFT utilizes a coarse-fine search strategy to improve the estimation accuracy. The coarse-fine strategy firstly obtains a crude estimation by searching the maximum FrFT coefficient of the received data, and then, the result is refined by modified methods such as Newton-type methods [8] and interpolation methods [10,18]. However, these methods require numerous extra calculations and make it difficult to handle the OFDM-LFM signals.

Inspired by the recently-developed fast iterative interpolated beamforming estimation method [19], we propose a fast and high accuracy estimator for uncooperative OFDM-LFM signals based on DFrFT and Fractional Autocorrelation (FA). We refer to the proposed method as Fast Iterative Interpolated DFrFT (FII-DFrFT), which exhibits desirable convergence properties and the same order computational complexity as Digital Fourier Transform (DFT).

The rest of this paper is organized as follows. In Section 2, the signal model of the intercepted uncooperative OFDM-LFM signal and the analytical formulations of DFrFT for this signal are given. In Section 3, the proposed FII-DFrFT is described. Section 4 gives the numerical simulation of the proposed algorithm, and some conclusions are drawn in Section 5.

## 2. Signal Model and FrFT

Let us consider a single-channel reconnaissance receiver and an adversary MIMO radar system with *M* transmitters. Assume that this radar employs OFDM-LFM waveforms, which were firstly introduced into the design of an MIMO radar system by F. Cheng [1]. Afterwards, the signal of the *m*-th transmitter is given as [1]:

(1) $$s_{m}\left(t\right)=u_{m}\left(t\right)e^{j2\pi f_{0}t},1\leq m\leq M$$

(2) $$u_{m}\left(t\right)=\frac{1}{\sqrt{T_{p}}}\mathrm{rect}\left(\frac{t}{T_{p}}\right)e^{j2\pi \left(mf_{\Delta }t+\frac{1}{2}\mu_{0}t^{2}\right)}e^{j\phi_{m}},1\leq m\leq M$$

where $f_{0}$ denotes the carrier frequency of the victim radar system, $T_{p}$ is the pulse duration, $f_{\Delta }$ is the frequency step between two adjacent transmitters, $\mu_{0}$ is the chirp rate and $\phi_{m}$ is the initial phase of the *m*-th transmitting signal. Here, we also assume $\mu_{0}T_{p}\ll f_{0}$ [1].

Therefore, the MIMO radar signal intercepted by the reconnaissance receiver can be written as:

(3) $$x\left(t\right)=A_{m}\sum_{m=0}^{M-1}s_{m}\left(t\right)+\omega \left(t\right)$$

where $A_{m}$ is the complex constant amplitude of the *m*-th subpulse and $\omega \left(t\right)$ represents zero-mean white Gaussian noise with variance $\sigma^{2}$. The receiver first detects the observed signal energy and estimates the carrier frequency. Here, it is assumed that the above steps have been accomplished [20,21]. Then, these detected pulse observations are demodulated into intermediate frequency and sampled at an appropriate frequency, $f_{s}$, which satisfies the bandpass sampling theorem. Thus, we can collect *N* successive samples of the signal pulse represented as:

(4) $$x\left[n\right]=A_{m}\sum_{m=0}^{M-1}e^{j2\pi \left[f_{m}nT_{s}+\frac{1}{2}\mu_{0}{\left(nT_{s}\right)}^{2}\right]}e^{j\phi_{m}}+\omega \left[n\right]$$

where $T_{s}=1/f_{s}$, $f_{m}=f_{I}+mf_{\Delta }$, $f_{I}$ denotes the demodulated intermediate frequency and $n=0,1,\cdots ,N-1\left(N=T_{p}f_{s}\right)$. The classical definition of FrFT [11] is:

(5) $$X_{\alpha }\left(u\right)=\int_{-\infty }^{\infty }K_{\alpha }\left(t,u\right)x\left(t\right)dt$$

where $K_{\alpha }\left(t,u\right)$ is the kernel function with:

(6) $$K_{\alpha }\left(t,u\right)=\left\{\begin{array}{cc} B_{\alpha }exp\left[j\pi \left(\left(u^{2}+t^{2}\right)\cot\alpha -2ut\csc\alpha \right)\right], & \alpha \neq k\pi \\ \delta \left(t-u\right) & \alpha =2k\pi \\ \delta \left(t+u\right) & \alpha =2\left(k+1\right)\pi \end{array}\right.$$

and $B_{\alpha }=\sqrt{\left(1-j\cot\alpha \right)}$. α=pπ/2 is called the rotation angle, while *p* is the order of FrFT. *u* is a spectral parameter. We employed the fast digital algorithm of FrFT [11], which is represented as:

(7) $$X_{\alpha }\left(\frac{U}{2\Delta x}\right)=\frac{B_{\alpha }}{2\Delta x}e^{j\pi \tan\left(\frac{\alpha }{2}\right){\left(\frac{U}{2\Delta x}\right)}^{2}}\sum_{n=-N}^{N}e^{j\pi \csc\alpha {\left(\frac{U-n}{2\Delta x}\right)}^{2}}e^{j\pi \tan\left(\frac{\alpha }{2}\right){\left(\frac{n}{2\Delta x}\right)}^{2}}x\left(\frac{n}{2\Delta x}\right)$$

where U=u2Δx and $\Delta x=\sqrt{N}$.

The OFDM-LFM signal is reformulated into multiple impulses only for a particular *p* in the FrFT domain, while the Gaussian white noise term is distributed evenly in the $\left(\alpha ,U\right)$ plane. After peak searching, the estimated coordinates $\left({\hat{\alpha }}_{0},{\hat{U}}_{m}\right)$ can be used to obtain the estimators for OFDM-LFM signal parameters as [10]:

(8) $$\left\{\begin{array}{c} {\hat{\mu }}_{0}=-\cot\left({\hat{\alpha }}_{0}\right)\frac{f_{s}^{2}}{N}\\ {\hat{f}}_{m}={\hat{U}}_{m}\csc\left({\hat{\alpha }}_{0}\right)\frac{f_{s}}{N}\\ {\hat{f}}_{\Delta }=\frac{1}{M-1}\sum_{m=2}^{M-1}\left(f_{m}-f_{m-1}\right)\\ {\hat{A}}_{m}=\frac{\left|X_{{\hat{\alpha }}_{0}}\left({\hat{U}}_{m}\right)\right|}{\Delta x\left|B_{{\hat{\alpha }}_{0}}\right|} \end{array}\right.$$

However, this estimation performance depends on the grid size used for searching, while the ideal impulses require that Equation (7) is computed on an infinite number of grid points. In practice, due to the finite sampling data and leakage of other components’ energy, there always exists some residual terms between the estimated quasi peaks $\left(\alpha_{B},U_{Bm}\right)$ and real peaks $\left(\alpha_{0},U_{m}\right)$, where $\alpha_{B}$ and $U_{Bm}$ represent the bias estimations. Here, we set the residual terms as $\delta_{0}$ and $\varepsilon_{m}$, where $\alpha_{0}=\alpha_{B}+\varphi_{0}$ and $U_{m}=U_{Bm}+\varepsilon_{m}$. Furthermore, we set $\varphi_{0}=\delta_{0}\Delta \alpha$, where Δα is the coarse searching interval of rotation α. In addition, it is reasonable to assume that $\delta_{0},\varepsilon_{m}\in \left[-0.5,0.5\right]$. Therefore, the residual term is the decisive point affecting the parameter estimation precision in Equation (8). Through conventional algorithms such as Newton-type [8] and interpolation [18], the residual term can be estimated. However, the first method suffers from a huge computational cost, and the second is only developed for monocomponent signals.

## 3. The Proposed Method

Our proposed estimation method is inspired by the multiple component estimator in [19], which was designed for direction of arrival estimation and implemented by DFT. However, if we want to use that idea in OFDM-LFM radar signal parameter estimation, some improvements on DFrFT should be conferred.

Substituting Equation (3) into Equation (5) and ignoring the noise term, the energy of the OFDM-LFM signal concentrates in the DFrFT domain:

(9) $$X_{\alpha_{0}}\left(U\right)=A_{m}B_{\alpha_{0}}e^{j\pi U^{2}\left(-\mu \right)}\sum_{m=0}^{M-1}\left\{e^{j\phi_{m}}\delta \left[2\pi \left(mf_{\Delta }-U\csc\alpha_{0}\right)\right]\right\}$$

After peak searching at a sufficient grid interval, Δα, we can obtain *M* peak coordinates $\left(\alpha_{B},U_{Bm}\right)$. Then, the true chirp rate and true center frequency of the *m*-th component is given by:

(10) $$\left\{\begin{array}{c} \mu_{0}=-\cot\left(\alpha_{B}+\delta_{0}\Delta \alpha \right)\frac{f_{s}^{2}}{N}\\ f_{m}=mf_{\Delta }=\left(U_{Bm}+\varepsilon_{m}\right)\csc\left({\hat{\alpha }}_{0}+\delta_{0}\Delta \alpha \right)\frac{f_{s}}{N} \end{array}\right.$$

In the following subsections, we will derive the estimator for chirp rate $\mu_{0}$ based on the $\delta_{0}$ and the estimator for center frequency $f_{m}$ based on the $\varepsilon_{m}$.

### 3.1. Estimator for Chirp Rate

Due to the fact that the analytical formulation of the quasi-peak amplitude $\left|X_{\alpha_{B}}\left(U_{Bm}\right)\right|$ in the FrFT domain involves the Fresnel integral formula [10], it is hard to construct the estimator for $\delta_{0}$ directly through the iterative method. Therefore, we introduce the FA algorithm to remove the Fresnel term, which is given as [22]:

(11) $$\left(x_{\alpha }^{*}x\right)\left(\tau \right)=\int x\left(t+\frac{\tau }{2}\sin\alpha \right)x^{*}\left(t-\frac{\tau }{2}\sin\alpha \right)e^{2j\pi t\tau \cos\alpha }dt$$

where τ represents the delay factor. Then, the FA envelope statistic is also given as:

(12) $$L\left(\alpha \right)=\int_{-\infty }^{\infty }\left|\left(x_{\alpha }^{*}x\right)\left(\tau \right)\right|d\tau$$

Substituting Equation (3) into Equations (11) and (12) and ignoring the noise term, we can derive the FA envelope of the OFDM-LFM signal:

(13) $$\begin{array}{cc} \left(x_{\alpha }^{*}x\right)\left(\tau \right) & =\int_{-\infty }^{\infty }\sum_{m=0}^{M-1}A_{m}s_{m}\left(t+\frac{\tau }{2}\sin\alpha \right)\sum_{m=0}^{M-1}A_{m}s_{m}\left(t-\frac{\tau }{2}\sin\alpha \right)e^{2j\pi t\tau \cos\alpha }dt\\ & =\int_{-\infty }^{\infty }\gamma \left(t\right)e^{j2\pi t\tau \left(\mu_{0}\sin\alpha +\cos\alpha \right)}\sum_{m_{i}=0}^{M-1}\sum_{m_{j}=0}^{M-1}A_{m_{i}}A_{m_{j}}e^{j\pi \tau f_{\Delta }\sin\alpha \left(m_{i}-m_{j}\right)}e^{j2\pi tf_{\Delta }\left(m_{i}-m_{j}\right)}dt \end{array}$$

(14) $$L\left(\alpha \right)=\int_{-\infty }^{\infty }\left|\left(x_{\alpha }^{*}x\right)\left(\tau \right)\right|d\tau$$

where $\gamma \left(t\right)=1/\sqrt{T_{p}}\mathrm{rect}\left(t/\sqrt{T_{p}}\right)$ and:

(15) $$\Gamma \left(\alpha \right)=\int_{-\infty }^{\infty }\int_{-\frac{T_{p}}{2}}^{\frac{T_{p}}{2}}\sum_{m_{i}=0}^{M-1}\sum_{m_{j}=0}^{M-1}A_{m_{i}}A_{m_{j}}e^{j\pi \tau f_{\Delta }\sin\alpha \left(m_{i}-m_{j}\right)}e^{j2\pi tf_{\Delta }\left(m_{i}-m_{j}\right)}dtd\tau$$

It is noticed that $\Gamma \left(\alpha \right)$ does not involve $\mu_{0}$; therefore, we can ignore it in the following analysis of this subsection. The real peak of $L\left(\alpha_{0}\right)$ satisfies $\mu_{0}=-\cot\alpha_{0}$. Substituting $\alpha_{B}=\alpha_{0}-\delta_{0}\Delta \alpha$ into Equation (14), we can obtain:

(16) $$\begin{array}{cc} L\left(\alpha_{B}\right) & =\int_{-\infty }^{\infty }\left|T_{p}\mathrm{Sinc}\left[\pi \tau \left(-cot\alpha_{0}\sin\left(\alpha_{0}-\delta_{0}\Delta \alpha \right)+\cos\left(\alpha_{0}-\delta_{0}\Delta \alpha \right)\right)\right]\right|d\tau \\ & =\int_{-\infty }^{\infty }\left|T_{p}\mathrm{sinc}\left[\pi \tau \csc\alpha_{0}\sin\left(\delta_{0}\Delta \alpha \right)\right]\right|d\tau \end{array}$$

When the searching interval Δα is small enough, it is reasonable to use the approximations $\sin\left(\delta_{0}\Delta \alpha \right)\approx \delta_{0}\Delta \alpha$ and $\sin\left[\pi \tau \csc\alpha_{0}\left(0.5-\delta_{0}\right)\Delta \alpha \right]\approx \sin\left[\pi \tau \csc\alpha_{0}\left(0.5+\delta_{0}\right)\Delta \alpha \right]$ in Equation (16). Then, we can construct the error mapping as:

(17) $$\beta =\frac{L\left(\alpha_{B}+0.5\Delta \alpha \right)+L\left(\alpha_{B}-0.5\Delta \alpha \right)}{L\left(\alpha_{B}+0.5\Delta \alpha \right)-L\left(\alpha_{B}-0.5\Delta \alpha \right)}\approx \int_{-\infty }^{\infty }\frac{\left|\frac{1}{\pi \csc\alpha_{0}\left(0.5-\delta_{0}\right)\alpha_{s}\tau }\right|-\left|\frac{1}{\pi \csc\alpha_{0}\left(0.5+\delta_{0}\right)\alpha_{s}\tau }\right|}{\left|\frac{1}{\pi \csc\alpha_{0}\left(0.5-\delta_{0}\right)\alpha_{s}\tau }\right|+\left|\frac{1}{\pi \csc\alpha_{0}\left(0.5+\delta_{0}\right)\alpha_{s}\tau }\right|}d\tau =\frac{1}{2\delta_{0}}$$

Hence, ${\hat{\delta }}_{0}=1/2\beta$ can be used as an estimator for $\delta_{0}$.

Finally, the new estimation of rotation angle $\alpha_{0}$ is presented as ${\hat{\alpha }}_{0}=\alpha_{B}+{\hat{\delta }}_{0}\Delta \alpha$. Then, by substituting $\alpha_{B}={\hat{\alpha }}_{0}$ and renewing ${\hat{\alpha }}_{0}$, an iterative method can be combined to improve the estimation accuracy.

### 3.2. Estimator for Center Frequency

Firstly, we consider the DFrFT for a monocomponent LFM signal. Substituting Equation (2) into Equation (7), we can obtain:

(18) $$X_{\alpha }\left(\frac{U}{2\Delta x}\right)=\frac{B_{\alpha }}{2\Delta x}e^{j\pi \cot\alpha {\left(\frac{U}{2\Delta x}\right)}^{2}}\sum_{n=-N}^{N}e^{j\pi 2n\left(\frac{f_{m}}{2f_{s}}-\frac{U\csc\alpha }{{\left(2\Delta x\right)}^{2}}\right)+j\pi n^{2}\left(\frac{k_{0}}{{\left(2f_{s}\right)}^{2}}+\frac{\cot\alpha }{{\left(2\Delta x\right)}^{2}}\right)}$$

According to the analysis in Section 3.1, we assume that ${\hat{\alpha }}_{0}\approx \alpha_{0}$. Hence, at the quasi peak $\left({\hat{\alpha }}_{0},U_{Bm}\right)$, Equation (18) can be approximated by:

(19) $$X_{{\hat{\alpha }}_{0}}\left(\frac{U_{Bm}}{2\Delta x}\right)=\frac{B_{{\hat{\alpha }}_{0}}}{2\Delta x}e^{j\pi \cot{\hat{\alpha }}_{0}{\left(\frac{U_{Bm}}{2\Delta x}\right)}^{2}}\sum_{n=-N}^{N}e^{j\pi n\left(\frac{f_{m}}{f_{s}}-\frac{U_{Bm}\csc{\hat{\alpha }}_{0}}{2N}\right)}$$

Using $f_{m}=U_{m}f_{s}\csc\alpha_{0}/2N$ and $U_{m}=U_{Bm}+\varepsilon_{0}$, we can rewrite Equation (19) as:

(20) $$X_{{\hat{\alpha }}_{0}}\left(\frac{U_{Bm}}{2\Delta x}\right)=\frac{B_{{\hat{\alpha }}_{0}}}{2\Delta x}e^{j\pi \cot{\hat{\alpha }}_{0}{\left(\frac{U_{Bm}}{2\Delta x}\right)}^{2}}\sum_{n=-N}^{N}e^{j\pi n\frac{\varepsilon_{0}\csc{\hat{\alpha }}_{0}}{2N}}$$

Similar to the approach in Section 3.1, we can obtain $X_{{\hat{\alpha }}_{0}}\left(\frac{U_{Bm}\pm P}{2\Delta x}\right)$ as:

(21) $$X_{{\hat{\alpha }}_{0}}\left(\frac{U_{Bm}\pm P}{2\Delta x}\right)=\Gamma^{\prime }\left({\hat{\alpha }}_{0},U_{Bm}\pm P\right)\left[\frac{e^{-j\pi \frac{\left(\varepsilon_{0}\mp P\right)\csc{\hat{\alpha }}_{0}}{2}}\left(1-e^{j\pi \left(\varepsilon_{0}\mp P\right)\csc{\hat{\alpha }}_{0}}\right)}{1-e^{j\pi \frac{\left(\varepsilon_{0}\mp P\right)\csc{\hat{\alpha }}_{0}}{2N}}}\right]$$

where:

(22) $$\Gamma^{\prime }\left({\hat{\alpha }}_{0},U_{Bm}\pm P\right)=\frac{B_{{\hat{\alpha }}_{0}}}{2\Delta x}e^{j\pi \cot{\hat{\alpha }}_{0}{\left(\frac{U_{Bm}\pm P}{2\Delta x}\right)}^{2}}$$

When $\left(\varepsilon_{0}\mp P\right)\ll N$, it is reasonable to use the approximation $1-e^{x}\approx x\left(x\to 0\right)$ in Equation (21). Then, by setting $P=1/\csc{\hat{\alpha }}_{0}$, we can construct the error mapping as:

(23) $$h=\frac{\left|X_{{\hat{\alpha }}_{0}}\left(\frac{U_{Bm}+P}{2\Delta x}\right)\right|+\left|X_{{\hat{\alpha }}_{0}}\left(\frac{U_{Bm}-P}{2\Delta x}\right)\right|}{\left|X_{{\hat{\alpha }}_{0}}\left(\frac{U_{Bm}+P}{2\Delta x}\right)\right|-\left|X_{{\hat{\alpha }}_{0}}\left(\frac{U_{Bm}-P}{2\Delta x}\right)\right|}=\frac{\varepsilon_{0}}{\csc\alpha_{0}}$$

Hence, ${\hat{\varepsilon }}_{0}=h\csc{\hat{\alpha }}_{0}$ can be used as an estimator for ${\hat{\varepsilon }}_{0}$. The fine estimation of $U_{m}$ is presented as ${\hat{U}}_{m}=U_{Bm}+{\hat{\varepsilon }}_{0}$. Then, by substituting $U_{B}={\hat{U}}_{0}$ and renewing ${\hat{U}}_{0}$, an iterative method can also be combined to improve the estimation accuracy.

### 3.3. Iterative Estimation for OFDM-LFM

In this subsection, we extend the proposed center frequency estimation method to the OFDM-LFM signals. The major difference between multiple center frequency estimation and single center frequency estimation is the estimation error that is caused by the leakage of multiple components in the OFDM-LFM signal. This error will lead to a bias in the interpolated DFrFT coefficients, deviating from their true values. Assuming the noise-free actual coefficients ${\tilde{X}}_{{\hat{\alpha }}_{0},m}\left(\left({\hat{U}}_{m}\pm P\right)/2\Delta x\right)$ of the *m*-th component, we obtain:

(24) $${\tilde{X}}_{{\hat{\alpha }}_{0},m}\left(\frac{{\hat{U}}_{m}\pm P}{2\Delta x}\right)=DFRFT_{\left({\hat{\alpha }}_{0},{\hat{U}}_{m}\pm p\right)}\left(x\left[n\right]\right)=X_{{\hat{\alpha }}_{0},m}\left(\frac{{\hat{U}}_{m}\pm P}{2\Delta x}\right)+\sum_{l=1,\left(l\neq m\right)}^{M}{\overset{⌣}{X}}_{{\hat{\alpha }}_{0},l}\left(\frac{{\hat{U}}_{m}\pm P}{2\Delta x}\right)$$

where ${\overset{⌣}{X}}_{{\hat{\alpha }}_{0},l}(\left({\hat{U}}_{m}\pm P\right)/2\Delta x)$ represent the leakage terms introduced by the other *M* − 1 OFDM-LFM components, which can be calculated by:

(25) $$\begin{array}{c} {\overset{⌣}{X}}_{{\hat{\alpha }}_{0},l}\left(\frac{{\hat{U}}_{m}\pm P}{2\Delta x}\right)=A_{l}\mathrm{DFrF}T_{\left({\hat{\alpha }}_{0},{\hat{U}}_{m}\pm p\right)}\left({\hat{s}}_{l}\left[n\right]\right)=A_{l}\frac{B_{{\hat{\alpha }}_{0}}}{2\Delta x}e^{j\pi \cot{\hat{\alpha }}_{0}{\left(\frac{{\hat{U}}_{m}\pm P}{2\Delta x}\right)}^{2}}\sum_{n=-N}^{N}e^{j\pi n\left(\frac{\left({\hat{U}}_{l}-{\hat{U}}_{m}\mp P\right)\csc{\hat{\alpha }}_{0}}{2N}\right)} \end{array}$$

where $A_{l}$ represents the complex amplitude of the *l*-th $\left(l=1,\cdots ,M\right)$ component in the fractional domain. Therefore, the estimation error of interpolated coefficients can be reduced by subtracting the sum of the leakage from other components, which is:

(26) $${\hat{X}}_{{\hat{\alpha }}_{0},m}\left(\frac{{\hat{U}}_{m}\pm P}{2\Delta x}\right)={\tilde{X}}_{{\hat{\alpha }}_{0},m}\left(\frac{{\hat{U}}_{m}\pm P}{2\Delta x}\right)-\sum_{l=1,\left(l\neq m\right)}^{M}{\overset{⌣}{X}}_{{\hat{\alpha }}_{0},l}\left(\frac{{\hat{U}}_{m}\pm P}{2\Delta x}\right)$$

Based on the above analysis, we propose an iteration-based algorithm to accomplish the parameter estimation of the OFDM-LFM signal, which is given in Algorithm 1.

**Algorithm 1:** Proposed fast iterative interpolated digital fractional Fourier transform method

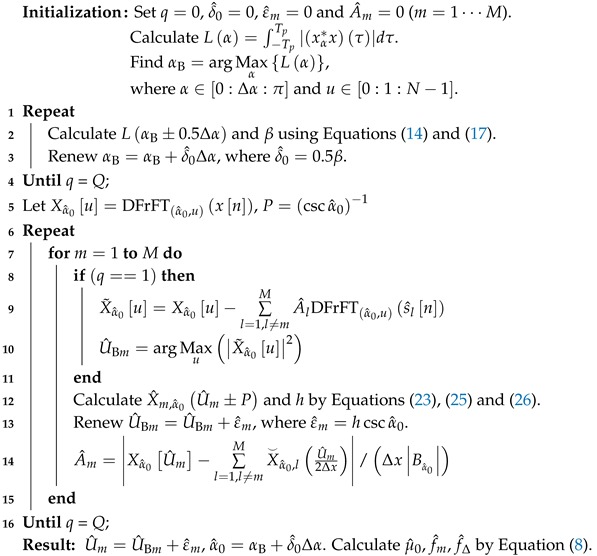



Next, we discuss the computational complexity of this method. The proposed method consists of two parts, which are the estimation process of $\alpha_{0}$ and the estimation process of $U_{m}$. The major computational load in the first part is due to the FA, which is about $O\left[G\left(2N\log N+N\right)\right]$ [22], where $G=\left\lfloor \pi /\Delta \alpha \right\rfloor$ ($\left\lfloor •\right\rfloor$ indicates the floor operator). The major computational load in the second part is due to the DFrFT during coarse searching, which is about $O\left(N\log N\right)$ [11]. In addition, there are *M*-times DFrFT coefficient calculations, whose computation complexity is about $O\left(2MN\right)$ during each iteration. Consequently, the overall complexity of the above-mentioned method can be expressed as approximately $O\left[GN\log N+N\log N+MN\right]$, which is more efficient than the methods in [6] (requires $O\left(GN^{2}\log N\right)$), [7] (requires $O\left(N^{3}\right)$) and [10] (requires $O\left(GN^{2}\log N\right)$), but less efficient than the method in [18] (requires $O\left(8N+N\log N\right)$).

## 4. Simulations

The goal of this section is to evaluate the estimation performance of the proposed method through Monte Carlo simulations. Consider two kinds of OFDM-LFM radar waveforms that are applied in different radar modes (searching and tracking modes). The employed simulation parameters are listed in Table 1, which are consistent with the simulation settings in [1]. As defined in [1], we consider that the signal amplitude $A_{m}$ of each subpulse is equal to $A_{0}$. As analyzed in Section 2, it is assumed that the signal detection and carrier frequency estimation have been accomplished. Here, we ignore the influence of signal detection probability and the accuracy of the carrier frequency estimation for the results. Then, the demodulated baseband pulse observations are sampled at the frequency $f_{s}=50\;\mathrm{MHz}$. In this context, the Normalized Mean Squared Error (NMSE) is used to evaluate the estimation accuracy. Furthermore, we define the Signal-to-Noise Ratio (SNR) as $\rho =10\mathrm{lg}\left(A_{1}^{2}/\sigma^{2}\right)$ and set the searching interval of rotation to be Δα=0.001. Some other algorithms reported in [6,7,10,18] and Cramer–Rao lower Bounds (CRB) [23] are also reviewed for comparison.

**Figure 1:**
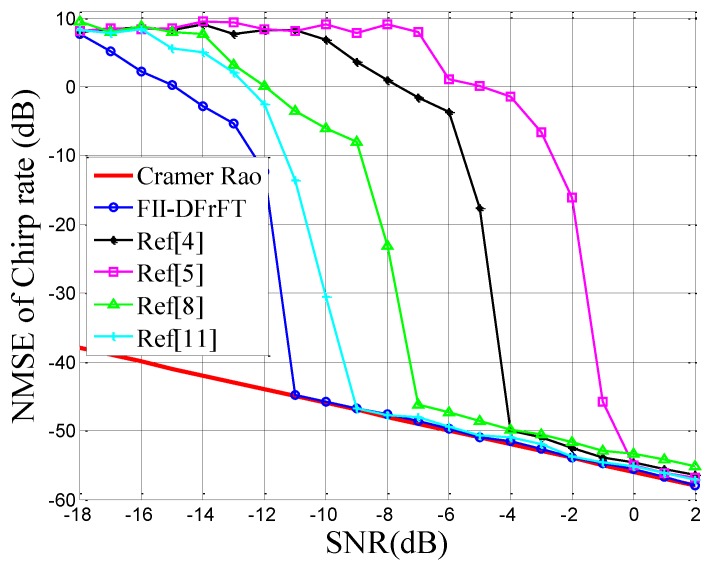
Normalized Mean Squared Error (NMSE) of $\mu_{0}$ for signal $\Omega_{1}$ versus the signal-to-noise ratio. FII, Fast Iterative Interpolated.

**Figure 2:**
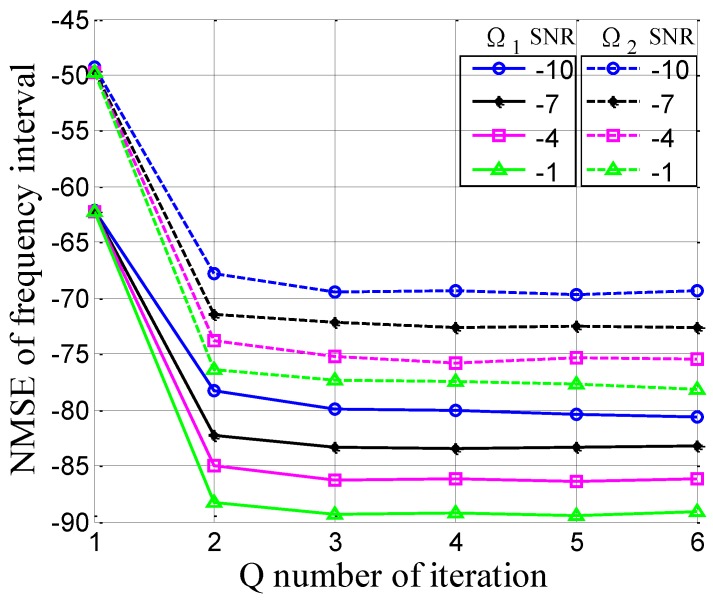
NMSE of $f_{\Delta }$ for signals $\Omega_{1}$ and $\Omega_{2}$ versus the number of iterations.

Figure 1 gives the NMSE of the chirp rate estimation, ${\hat{\mu }}_{0}$, versus different SNRs. In this simulation, Monte Carlo experiments were repeated 500 times for each SNR from −18 dB to 2 dB. It is obvious from Figure 1 that most NMSE curves of estimation algorithms approach or achieve the CRB at specific SNRs. Among them, the performance of the proposed method coincides with the CRB at the lowest SNR, which is −11 dB. Moreover, the simulation result from Figure 1 confirms that the estimation performance of the proposed method slightly outperforms the other algorithms at all SNRs. Here, the iteration number is set to Q=3, which is demonstrated in Figure 2.

In Figure 2, we study the effect of the iteration number, *Q*, on the convergence characteristics of the proposed method. In this simulation, the NMSE curves of frequency step ($f_{\Delta }$) estimation versus the iteration number (*Q*) when the SNR is set to $\left[-10,-7,-4,-1\right]\;\mathrm{dB}$ are depicted. Here, both signals with parameters from $\Omega_{1}$ and $\Omega_{2}$ are used for the simulation. As can be seen, the parameter estimation performance converges after three iterations for almost all of the SNRs. Therefore, through the simulation in Figure 2, the iteration number *Q* is suggested to be chosen as three.

## 5. Conclusions

In this study, we have derived the analytical AF and DFrFT approximation of OFDM-LFM radar signals. A new method called FII-DFrFT was proposed for uncooperative OFDM-LFM parameter estimation, which was formulated by locating the bottleneck issue that affects the estimation performance. The analytical formulas were hence derived, as well as their performance evaluation. Numerical simulations showed the validity and superiority of the proposed method, through comparisons with some existing algorithms at different SNRs. Nevertheless, as an uncooperative facility, especially for hostile MIMO radars, the estimation performance in the presence of clutter and other structure interferences is still a challenge for most cases. Hence, in future research, we would like to focus on the derivation and evaluation, taking into consideration the keen factors’ uncertainty, as well as the clutter background, before the proposed scheme is employed for practical applications.

## Figures and Tables

**Table 1:** Parameters of Orthogonal Frequency Division Multi-Linear Frequency Modulation (OFDM-LFM) signals.

	$\Omega_{1}$	$\Omega_{2}$
Radar operation mode	Searching	Tracking
Number of antennas *M*	4	4
Pulse duration $T_{p}$	20 μs	20 μs
Chirp rate $\mu_{0}$	0.15 MHz/μs	0.15 MHz/μs
Bandwidth $B_{0}$	3 MHz	3 MHz
Frequency step $f_{\Delta }$	5 MHz	1.5 MHz

## Abbreviations

The following abbreviations are used in this manuscript:

OFDM	Orthogonal Frequency Division Multi-
LFM	Linear Frequency Modulation
FrFT	Fractional Fourier Transform
FA	Fractional Autocorrelation
MWHT	Multiple Wigner–Hough Transform
DFrFT	Digital FrFT
DFT	Digital Fourier Transform
NMSE	Normalized Mean Squared Error
SNR	Signal-to-Noise Ratio
CRB	Cramer–Rao lower Bound
